# Haptic Sensing and Feedback Techniques toward Virtual Reality

**DOI:** 10.34133/research.0333

**Published:** 2024-03-23

**Authors:** Yuxiang Shi, Guozhen Shen

**Affiliations:** ^1^ School of Integrated Circuits and Electronics, Beijing Institute of Technology, Beijing 100081, China.; ^2^ Institute of Flexible Electronics, Beijing Institute of Technology, Beijing 102488, China.

## Abstract

Haptic interactions between human and machines are essential for information acquisition and object manipulation. In virtual reality (VR) system, the haptic sensing device can gather information to construct virtual elements, while the haptic feedback part can transfer feedbacks to human with virtual tactile sensation. Therefore, exploring high-performance haptic sensing and feedback interface imparts closed-loop haptic interaction to VR system. This review summarizes state-of-the-art VR-related haptic sensing and feedback techniques based on the hardware parts. For the haptic sensor, we focus on mechanism scope (piezoresistive, capacitive, piezoelectric, and triboelectric) and introduce force sensor, gesture translation, and touch identification in the functional view. In terms of the haptic feedbacks, methodologies including mechanical, electrical, and elastic actuators are surveyed. In addition, the interactive application of virtual control, immersive entertainment, and medical rehabilitation is also summarized. The challenges of virtual haptic interactions are given including the accuracy, durability, and technical conflicts of the sensing devices, bottlenecks of various feedbacks, as well as the closed-loop interaction system. Besides, the prospects are outlined in artificial intelligence of things, wise information technology of medicine, and multimedia VR areas.

## Introduction

Virtual reality (VR) is a novel technique that can combine input/output data and instructions, including visual, auditory, olfactory, haptic, and gustatory information, into an organic whole. Its real-time responding to outside input from operator and simultaneously adjusting inner output to act on operator make it a closed-loop human machine interaction (HMI) [1]. Haptic interaction plays an important role in VR system for its realistic interactions between operator and the virtual object [2,3], which contributes to more immersive virtual experiences, and is also worthy of applications such as virtual training and virtual controlling.

Haptic sensation is defined as acquiring information in the process of contact or operation via skin tactile system, which is supposed to be the most complex sensation because it relies on the tactile receptors distributed on the whole body. The density of tactile cells under epidermis is about 100/cm^2^ [4], which can detect various kinds of outside stimuli. This complete sensing system is also the perfect to be imitated by artificial tactile interfaces in HMI area.

From technical aspect, haptic interaction mainly contains tactile sensing and haptic feedback. Tactile sensing devices are capable of acquiring touch characteristics via proximity or contact between human hand and object. Tactile sensor has two prime functions of sensing and recognition. The first is to detect the contact conditions including contact or not, touch area, pressure value, pressure distribution, and even multidimensional force [5,6]. Other information, such as the physical characters of object (e.g., smoothness, hardness, texture, and shape) and operation status (e.g., the contact, friction, and slippage), can also be collected by tactile sensors [7–9]. This function can real-time monitor the interaction process between robot hand and operand, which is beneficial for the efficient virtual control or robot teleoperation [10–12]. The second function of tactile sensor is smart identification containing gesture capturing and texture identifications [13–15]. For example, the gesture recognition by tactile sensor array has been applied in extracting hand movement as input instructions in virtual control area, and the captured gesture information are essential data to model virtual object in motion-sensing entertainment. In addition, the texture, stiffness, and material identification are also emerging technologies that have improved the versatility of tactile sensors in teleoperation and VR system.

Haptic feedback, a reverse process of tactile sensing, is to stimulate skin to evoke the tactile receptor and generate tactile sensations via feedback devices just like touching a real object [16–18]. It is an interface aiming to enable bilateral signal communications between human and computer [19,20], and is indispensable for enhancing immersion, interaction, and imagination of VR system. Haptic feedback device has been applied in virtual training, virtual surgery, and dynamic braille, and it is considered as the most important component than virtual vision and audition, because the real-time control and feedback is necessarily dependent on these tasks.

**Fig. 1.**
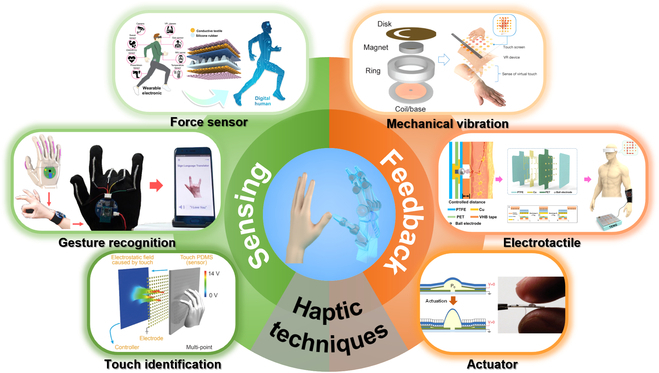
Haptic sensing and feedback techniques. Reproduced with permission from [88,101,114,136,147,155]. Copyright, Springer Nature, John Wiley and Sons, and the American Association of the Advancement of Science.

In this review, to address the importance of tactile sensor and haptic feedback shown in Fig. 1, we primarily elaborate their advanced techniques and versatile applications toward HMI in VR areas. We first introduce the sensing mechanisms of human haptics from anatomic aspect with details of various tactile receptors (in the “Fundamentals of Human Haptics” section), which is the guidelines for imitating the advanced skin tactile systems to design multifunctional haptic sensors and gives details of haptic for the feedback device to selected appropriate parameters (e.g., the blocking force, frequency, and displacement) to stimulate skin. Then, in the “Tactile Sensing Devices” section, tactile sensors in VR system are presented with force sensor, gesture recognition, and touch identification. The haptic feedback techniques are recommended in the “Haptic Feedback Techniques” section, covering specific feedback types of mechanical vibration, electrotactile (ET) display, and dielectric elastomer actuator (DEA). Furthermore, the applications of these devices, such as virtual input, force mapping, immersive entertainment, and feedback prosthesis, are summarized in the “Applications” section. We finally review the challenges to develop tactile interfaces in VR system, and the potential opportunities are also expected in the end.

## Fundamentals of Human Haptics

Tactile sensation is a synergetic effect of various neuro-system responding to press, temperature, and joint position. It can sense the microcosmic/macroscopic and spatial characteristics of the stimulus. The microcosmic features include material, roughness, and viscosity, while macroscopic characters cover shape, size, and structure and the space information relates to the special position of the stimuli. When object of different characters contacts with skin, skin can generate various encoded tactile information with various receptors under skin.

**Fig. 2.**
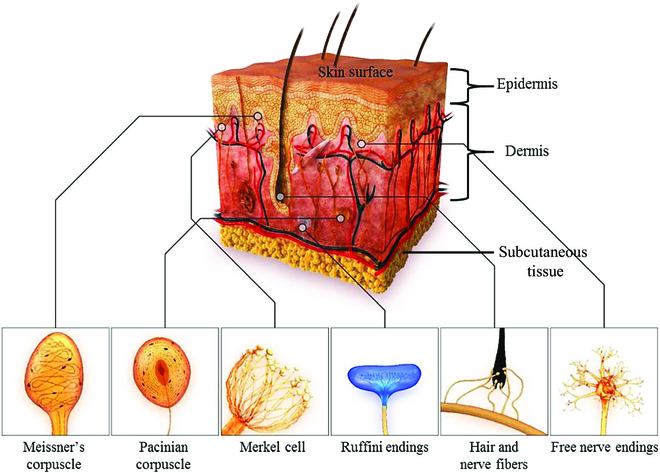
Mechanoreceptors in human skin. Reproduced with permission from [21]. Copyright, 2018, MDPI.

Tactility is mainly realized by human skin. Skin is not only the first line of defense of human body but also the important organ for brain to sense outside stimuli and make fast response. It is the most direct and fundamental approach for human communicating with the outside world. The structures of skin are shown in Fig. 2 [21]. Human skin consists of epidermis, dermis, and subcutaneous tissue. The fine hair locates across the epidermis and dermis, and roots in the follicles at dermis. Quantities of nerve endings distributed around the fine hair and those with tactile sensing abilities are mechanoreceptors. The mechanoreceptor can generate neuro-signals when stimulating the epidermis and transmit signals to brain neuro-systems. These mechanoreceptors are mainly classified into four categories: Merkel cells, Meissner corpuscles, Ruffini endings, and Pacinian corpuscles [22–25]. Each receptor only shows exclusive response to specific mechanical stimulus, and these responding processes are heavily depended on the mechanical character of the local skin. From the adaptation rate aspect, these mechanoreceptors can be categorized into two units: slow adapting (SA) receptors and fast adapting (FA) receptors. FA receptors can generate frequent output responding to dynamic stimulus, while SA receptors respond to static stimulus with time-invariant signals. Furthermore, in terms of the area of receptive field and density, each unit covers type I and type II receptors, among which the type I receptors distribute closely to the epidermis and have small receptive fields, while type II receptors are at the deeper dermis and have wide receptive fields. The Merkel cell belongs to SA-I mechanoreceptor, is located approximately 1.5 mm from the epidermis, and is very sensitive to static pressure and the stimuli of object’s surface structure. Ruffini corpuscle, an AS-II mechanoreceptor at deep dermis, is responsible for dynamic mechanical stimulus and is sensitive to skin stretch and slips especially at fingertips. The FA-I type receptor, Meissner corpuscle, at the 0.5 to 0.7 mm close to the epidermis, shows the highest sensitivity to low frequent vibration of 10 to 50 Hz. It can detect light contact (e.g., tapping) and is in charge of the grip control and texture identification. The last one Pacinian corpuscle is FA-II type receptor at depth 1.5 to 2 mm in skin, which is responsible for sensing vibrations at frequency from 200 to 300 Hz, which is very crucial to percept surface textures. Details of the four receptors are summarized in Table 1. The synergistic operation of the four receptors in various modes can impart human hand with abilities to finish tasks more precisely and delicately than any advanced robotic system. Analyzing the mechanoreceptor in skin is beneficial for precisely understanding the generation of tactile sensation and is worthy of mimicking tactile sensors and designing haptic feedback devices.

**Table 1.** Characteristics of the four main mechanoreceptors in human skin. Reproduced with permission from [21,184]. Copyright, 2018, MDPI.

	Meissner corpuscle	Pacinian corpuscle	Merkel cell	Ruffini endings
Category	FA-I	FA-II	SA-I	SA-II
Adaptation rate	Fast	Fast	Slow	Slow
Location	Shallow	Deep	Shallow	Deep
Stimuli frequency (Hz)	10–200	70–100	0.4–100	0.4–100
Density (units/cm^2^)	140	30	70	10
Spatial resolution (mm)	3–4	>10	0.5	>7
Functional range	Object slip, light contact, texture	High-frequency, vibrations	Static forces with high resolutions	Tension deep in the skin and fascia
Receptive field (RF)	Small and sharp, 3–5 mm	Very large and diffuse, >20 mm	Small and sharp, 2–3 mm	Large and diffuse, 10–15 mm

## Tactile Sensing Devices

**Fig. 3.**
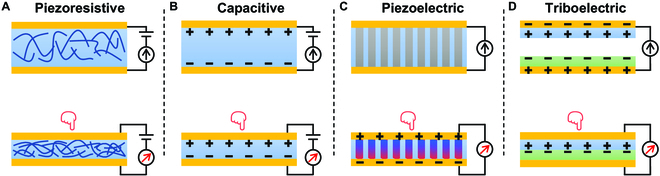
Mechanisms of four classic haptic sensing devices. (A) Piezoresistive. (B) Capacitive. (C) Piezoelectric. (D) Triboelectric.

Tactile sensors in skin can detect interactive information generated from physical contact between skin and environment. Simulating this function of human skin, artificial tactile sensors are invented to gather haptic information, such as physical contact and mechanical sliding, and contact-based details including material, roughness, and texture. In VR system, tactile sensor is the interface between human and machine, monitoring the contact station between the two sides. With the development of main sensing mechanism including piezoresistive [26–28], piezoelectric [29,30], capacitive [31,32], and triboelectric [33–35] (Fig. 3A to D), tactile sensor has advanced from force sensor [36,37] to gesture recognition [38,39] and even intelligent identification [40,41].

Piezoresistive haptic sensor relies on the changed resistance with mechanical stimuli to realize mechanoelectrical transduction, and the main mechanism is summarized by the equation of resistance

$$R=\frac{\rho L}{A}$$

where *ρ* is the resistive, and *L* and *A* are the length and cross-sectional area of the resistor device, respectively. The signal originates from the changed macroscopic geometrical parameter (*L* and *A*), where *L* increases and *A* decreases because of the Poisson effect upon stretching state [42], and also the changed *ρ* resulting from the dynamic percolation or quantum tunneling [43] of the conductive filler in dielectric layer [such as silver nanowires (Ag NWs)] or varied energy band structure of semiconductors (such as carbon nanotubes [44], graphene [45], or MXene [46]). Piezoresistive sensors are popular for high sensitivity, simple readout circuits, and low signal-to-noise ratio, but sometimes suffer from unfortunate frequency response due to hysteresis.

Capacitive sensors are always sandwiched structure with a dielectric layer between two electrode layers, and the mechanoelectrical signal is determined by

$$C=\frac{\varepsilon_{0}\varepsilon_{r}A}{d}$$

where *ε*_*0*_ and *ε*_*r*_ are the permittivity of free space and the relative permittivity of the dielectric layer, respectively, and *A* and *d* represent the effective overlapping area and distance of the two electrodes, respectively. The sensitivity of capacitive sensor depends on the changes of *A* and *d* to external mechanical stimuli, where *A* is sensitivity to shear force and *d* responds to the normal pressure. The sensitivity of capacitive sensor can be improved by embedded fillers (e.g., ionic liquid [47] and Ag NWs [48]) and specific microstructures (such as micropyramid [49] and micropillar [50]). Although capacitive sensors are sensitive to electromagnetic stimuli, they merit attention for the high sensitivity, low power consumption, and temperature interferences.

Piezoelectric sensor originates from the piezoelectric effect that the dipole separation of piezoelectric materials is able to generate electric field due to the deformation under mechanical stimuli. The first piezoelectric effect comes from the shift of anions’ and cations’ center in noncentrosymmetric crystal structure materials, for example, zinc oxide (ZnO) [51], lead zirconate titanate (PZT) [52], aluminum nitride (AlN) [53], and cadmium sulfide (CdS) [54]. Another mechanism is the re-alignment of permanent dipole moment in material, such as the poled polyvinylidene fluoride (PVDF) [55]. Piezoelectric constant, *d*_33_, is induced to quantify the capability of materials to transduce mechanical signal into piezoelectric potential. Inorganic materials, PZT, ZnO, and CdS, are sensitive to mechanical loads because of high *d*_33_, but limited in high Young’s modulus that leads to poor performance in haptic sensing. PVDF is naturally flexible, but its *d*_33_ is low. Thus, the combination of polymer matrix and inorganic piezoelectric material has been the effective approach to trade off the dielectric and mechanical properties [56]. The instantaneous electrical signal of piezoelectric sensor responding to high frequency load makes it an ideal candidate for measuring vibration.

Triboelectric sensors work on triboelectric effect and electrostatic induction, which can convert irregular low-frequency mechanical stimuli into electrical signal. As shown in Fig. 3D, the triboelectric effect brings positive and negative electrostatic charges on the two tribomaterials, while electrostatic induction causes current flow between the external circuit linking the two back electrodes. Triboelectric sensors possess a wide range selection of materials, such as polytetrafluoroethylene (PTFE), fluorinated ethylene propylene (FEP), polyimide (PI), and polyvinyl chloride (PVC) as positive triboelectric materials and polyamide (PA), polyurethane (PU) Cu, cotton, indium tin oxide, and human skin as negative triboelectric materials [57]. The electrostatic charges can be generated from friction [58], corona polarization [59], and iron irradiation [60]. Although poor signal-to-noise ratio causes difficulties, the diverse working mode [33], high sensitivity under low frequency, and self-powered naturality make it promising in haptic HMI areas.

### Force sensor

Pressure measurement is the most basic function of tactile sensors by converting force input into electrical signals. The sensing signal can be simply recorded as “0” or “1” to represent contact or contactless station [61,62], and the force quantity can also be characterized with correction by standard curve [63,64]. Choi et al. [65] presented a transparent and linear capacitive pressure sensor with high linearity (*R*^2^ = 0.995) over a wide pressure range (5 to 100 kPa). Serving as a transparent cover on phone screen, it could directly convert mechanical touch into electrical signal to input contact signal and even record the sliding trajectory, showing promising application as input unit in a VR system. Bai et al. [66] reported an iontronic pressure sensor with ultra-broad-range (0.008 Pa to 360 kPa) high sensitivity. They induced graded intrafillable architecture strategy that boosted the sensitivity and simultaneously widened the pressure sensitivity. With the correction of standard curve, it could respond to a broad pressure range of 360 kPa with a sensitivity over 200 kPa^−1^. The integrated microsensor array also presented high resolution under submillimeter of 100 μm with negligible noise, which merited tactile sensors with high sensitivity under wide pressure range.

**Fig. 4.**
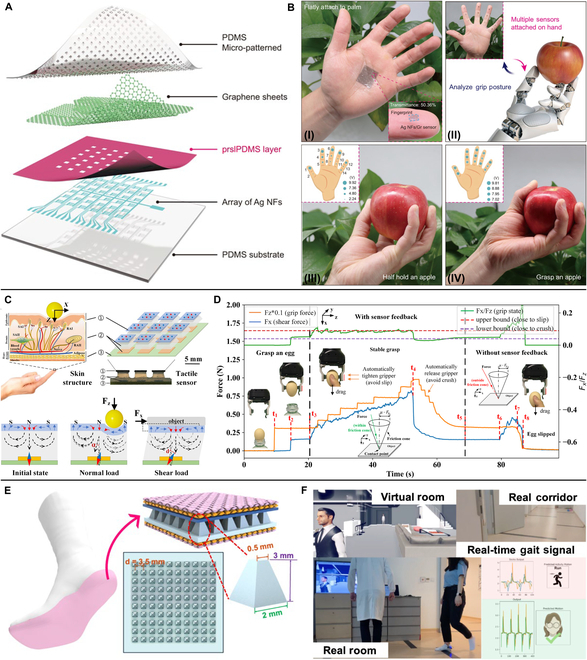
Tactile sensing devices of force sensors for VR applications. (A and B) Schematic illustration of the structure of pressure sensor arrays with micro-cage structures (A) and its application process (B). (I) Photographs of sensor arrays attached on palm. (II) Multiple sensors attached to the front of the finger to detect the different grasping postures. The voltage signal of the sensors at fingertip with a half hold apple (III) and fully grasped (IV). Reproduced with permission from [72]. Copyright 2023, Springer Nature. (C and D) Illustration of skin-inspired soft magnetic tactile sensor with working principle (C) and its application for an egg-grasping task (D). Reproduced with permission from [82]. Copyright 2021, the American Association for the Advancement of Science. (E and F) Schematic illustration of the intelligent sock sensor for monitoring plantar pressure (E) and the established digital human system with the sock senor (F). Reproduced with permission from [88]. Copyright 2020, Springer Nature.

For force sensor, the high resolution under linear response is always the target for researcher in area of tactile sensing. So far, various processing strategies have been explored to fabricate the active layer in force sensors, for example, laser microprocessing [67], photolithography [68], 3D printing [69], and textile technology [70]. Huang et al. [71] developed a paper-based force sensor with embedded Ag NW micro-probe arrays. Through double-sided laser printing approach, Ag NWs were fabricated on paper substrate with 2.5-mm unit size, and the sensor array could sensitively detect spatial distribution of touch pressure. As shown in Fig. 4A, Zhang et al. [72] induced photolithography strategy to design a flexible pressure sensor array with ultralow spatial cross-talk. Silver nanofibers (Ag NFs) and polydimethylsiloxane (PDMS) were etched with micropatterns and integrated with a photo-reticulated strain localization film (prslPDMS) to form a sensor array with micro-cage structure. Thus, this sensor array exhibited reduced pixel deformation overflow by 90.3%, sufficient pressure resolution to detect 1 g (~150 Pa) even in bend condition, and simulated pixel resolution over 4,000 ppi. Assembled on phalanges of the palm, it could monitor the pressure on fingertip when grasping an object (Fig. 4B), showing its ability in HMI. Zhang et al. [73] produced force sensors via a fast photocurable, solid-state conductive ionoelastomer (SCIE) by high-resolution three-dimensional (3D) printing. The printed SCIE-based building blocks had high-resolution architectures (about 50-μm overhanging lattices), high Young’s modulus, good stretchability, and sustained conductivity in a broad temperature range. It could be shaped into 3D flexible tactile sensors with enhanced performance, such as the printed gyroid-based piezoresistive sensor exhibiting sensitivities of 3.7-fold higher than its bulky counterparts. Qi et al. [74] reported a kind of sensing yarn based on core-shell fibers of stretchable electrode and piezoresistive elastic wrap. The textile structure endowed the sensor with high sensitivity (12.3 N^−1^) with wide sensing range (0.001 to 5 N), relatively larger contact area multiple contact sites, and larger deformation space for multimodal mechanical stimuli, which offered tactile sensors potentialities in wearable VR components, and artificial intelligence applications. Huang et al. [75] proposed a flexible capacitive sensor based on PDMS microfoam and laser-induced graphene electrode. The sandwiched structure of plate–foam–plate provided high sensitivity of about 0.026 kPa^−1^ in 15 to 40 kPa with low hysteresis of about 9.762%. Accordingly, this sensor could be integrated on gloves to detect joint movements and was possible to be applied in gesture recognition. The iontronic sensor reported by Luo et al. [76] was based on slant hierarchical microstructure inspired by gecko. This hierarchical layer, acting as electrode contacting with an ionic gel layer, could eliminate the pressure resistance and increase the functional interface expansion, which improved the relative capacitance change in both the low- and high-pressure region and realized the sensitivity of 36,000 kPa^−1^ and effective measurement range up to 300 kPa.

With the demand for detecting complex mechanical stimuli, tactile sensors are gradually optimized to respond to multidimensional forces such as pressure [77], shear force [78], and twisting force [79]. Chen et al. [80] proposed a skin-inspired multidimensional sensor capable of detecting 3D stimuli in three orthogonal axes. The sensor was rationally integrated into three subsensors of highly anisotropic, which realized distinguishing various mechanical stimuli including in-plane tension, normal pressure, and shearing. Ren et al. [81] reported a fully elastic and metal-free tactile sensor that could detect both normal and tangential forces. The elastic deformation was realized by tiny burr arrays on the active layer. Thus, the sensitivity of the sensor responding to normal pressure was improved to 51.43 kPa V^−1^, and a wide detection limit of tangential force from 0.3 to 40 N with rough sensitivity of 0.83 N V^−1^ (0.5 to 3 N) and 2.50 N V^−1^ (3 to 40 N) was achieved. As shown in Fig. 4C, Yan et al. [82] demonstrated a soft tactile sensor with self-decoupling and super-resolution abilities based on a sinusoidally magnetized flexible film. Its deformation could be characterized by a Hall sensor based on the varied magnetic flux densities under external forces. Accordingly, this sensor could accurately measure normal and shear force with a single unit and realize a 60-fold super-resolved accuracy enhanced by deep learning. The well performance of this sensor is shown in Fig. 4D, assisted by the sensor, and the robotic gripper could accomplish challenging tasks such as stably grasping fragile objects, which was beneficial for VR-based HMIs.

Besides abovementioned force sensors, plantar pressure monitoring is also an important aspect in VR system [83–87]. For example, as shown in Fig. 4E, Zhang et al. [88] produced a kind of intelligent socks based on triboelectric effects for monitoring foot-based activities. Frustum structure of millimeter scale was patterned on silicone rubber aided by 3D-printed mold to design the active layer, and the integrated socks could harvest waste energy from low-frequency body motions to transmit wireless sensory data. It could help to gait analysis realizing a 93.54% identification accuracy of 13 participants and recognize five human activities with high accuracy (96.67%). In this case, a digital human system was established (Fig. 4F) for applications in healthcare monitoring, personal identification, and future smart home. Shi and colleagues [89] reported a smart floor monitoring system based on triboelectric sensor. It is fabricated with unique “identity” electrode patterns using scalable screen printing technique and enabled a parallel connection to reduce system complexity as well as the deep-learning computational cost. The developed smart floor technology exhibited its potentiality in establishing virtual building/home system in near future.

### Gesture recognition

Gesture can convert physical motions into digital language and is capable of delivering ideas, emotions, and decisions [90]. In VR system, gesture recognition enables human to realize gesture navigation and virtual control close to real world, resulting in a more direct HMI approach, which is also necessary for building a more immersive virtual real world [91,92]. Hence, it means to precisely reproduce hand or body gesture in a computer-created virtual space. The first key segment of this process is sensing techniques [91–95]. Tactile sensors are able to respond to stretching and bending movement of hand or body motions and have been applied in gesture recognition in recent years [96–99].

**Fig. 5.**
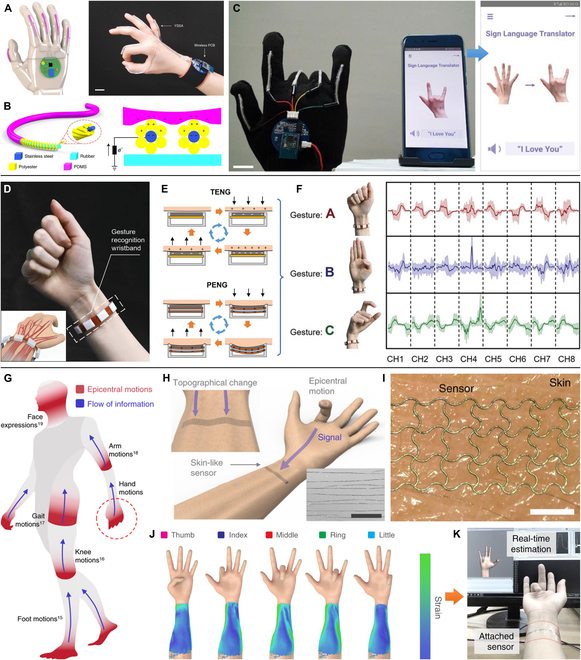
Tactile sensing devices of gesture recognitions for VR applications. (A to C) Schematic illustration of the triboelectric yarn sensor array (A), working principle (B), and the wearable sign-to-speech translation system (C). Reproduced with permission from [101]. Copyright 2020, Springer Nature. (D to F) Photograph of the gesture recognition wristband sensor (D), working principle (E), and gesture data processing (F). Reproduced with permission from [102]. Copyright 2022, John Wiley and Sons. (G to K) Schematic depicting the possible flow of body information with the epicentral sensor (G), illustration of measuring the epicentral motions of fingers (H) with the insert scanning electron microscopy (SEM) image of the cracked sensor (scale bar, 40 μm), photograph of the sensor conformably on skin (I), depiction of skin deformations for different finger bending motions (J), and photograph of the system for gesture duplication (K). Reproduced with permission from [104]. Copyright 2020, Springer Nature.

Wang et al. [100] proposed bioinspired data fusion architecture based on skin-like flexible strain sensor made of single-walled carbon nanotubes. The somatosensory data from sensor integrated with visual data were processed by the architecture and performed the gesture recognition. They finally built an auto-recognition and feedback system, which realized gesture-controlled quadruped robot, showing the potential application in virtual control. As shown in Fig. 5A and B, Zhou et al. [101] designed a wearable sign-to-speech translation system-based triboelectric yarn sensor array. Owing to unique structure and induced soft materials, the yarns are self-adaptive to fingers no matter under releasing or stretching condition. Mechanical motions of fingers could be converted into electrical signals via the sensor arrays, and assisted by deep learning, electric signals were processed to realize sign-to-speech translation. Figure 5C exhibits the wireless real-time translation system, and a total of 660 sign language hand gestures (American Sign Language) were successfully analyzed with a high recognition rate of 98.63% and a short recognition time of less than 1 s.

Tan et al. [102] reported a gesture recognition wristband that could achieve virtual keyboard input (Fig. 5D). As the inset in Fig. 4D shows, this wristband was based on physiological anatomy to capture movements of the muscle belly or tendon in wrist that was related to hand activities. Relying on the hybrid signal of piezoelectric and triboelectric sensor (Fig. 5E), this wristband could precisely recognize mechanical information regarding hand gesture without consuming electricity (Fig. 5F), and a maximum accuracy of 92.6% in recognizing 26 letters is achieved. Thus, it was considered to be applied in decoding gesture command and sign language translation, which could broaden the input modalities of VR systems. Moin et al. [103] proposed a wearable biosensing system with in-sensor adaptive machine learning for hand gesture. The main part of this system was a screen-printed, conformal electrode array that could collect electromyography signals, and it was characterized with in-sensor adaptive machine learning abilities. The reported experiments proved its performance that hand gesture classification accuracy of 97.12% was obtained when classifying 13 hand gestures using only a 4-s window of training data per gesture.

Figure 5 (G to K) shows a deep-learned skin sensor developed by Kim et al. [104] that could decode the epicentral human motions. This sensor was fabricated by consecutive laser serpentine patterning electrode allowing to conformably attach to epidermis (Fig. 5H and I), and high sensitivity was achieved with the laser-induced nanoscale cracking. The sensor system was capable of collecting data from arbitrary part of the wrist (Fig. 5J) and automatically training the model in real-time demonstration with a virtual 3D hand that reproduced real hand motions (Fig. 5K). Besides, it was also available for pelvis to capture dynamic gait movements in real time and would facilitate an in-direct remote measurement of human movements that contributed to VR applications.

**Fig. 6.**
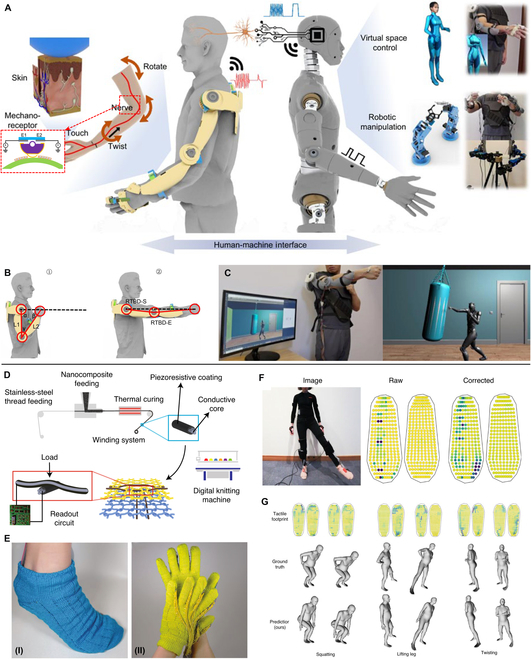
Tactile sensing devices of body movements recognition for VR applications. (A to C) Schematic illustration of the exoskeleton sensory system for realizing the manipulation in virtual space and robotics (A), body kinetic analysis (B), and its application in controlling a virtual figure for immersive physical training (C). Reproduced with permission from [105]. Copyright 2021, Springer Nature. (D to G) Schematic manufacturing method of the scalable piezoresistive fibers (D), the fabricated textile sensor (E), the readouts of plantar pressure from the sock sensor (F), and various poses recorded by the sensor system (G). Reproduced with permission from [26]. Copyright 2021, Springer Nature.

Zhu et al. [105] designed an exoskeleton manipulator based on bidirectional triboelectric sensors that could recognize body motions. As shown in Fig. 6A, this exoskeleton could capture movements of arm and fingers and then project these into robotic arm or virtual space. Because of the structural consistency between the exoskeleton and the human body, further kinetic analysis offered additional physical parameters without introducing other types of sensors (Fig. 6B). Figure 6C demonstrates its application in controlling a virtual figure for immersive physical training. Guo et al. [106] reported a wearable multidimensional motion sensor toward VR sports based on detecting both vertical and planar movements. This sensor could be integrated with a belt to identify low degree-of-freedom motions. Assembled on ankle position, it was able to achieve differentiated kicking force and direction with an accuracy of 97.5%. Thus, the virtual game (e.g., fitness game and shooting game) was successfully demonstrated. Luo et al. [26] fabricated a kind of conformal tactile textile to integrate a tactile learning platform. Figure 6D depicts the fabrication method of the piezoresistive fibers, and the textile sensor was able to be assembled on various parts of clothing (Fig. 6E). This platform could estimate whole-body gestures according to the distribution of the plantar pressure (Fig. 6F and G) contributing to the promising wearable VR systems.

### Touch identification

Aiming to establish more immersive VR systems, functions of tactile sensors are not limited in force and motion recognitions, but extended to intelligent identifications [107–109], such as pressure mapping [110,111], texture[112] , and object recognition [113].

**Fig. 7.**
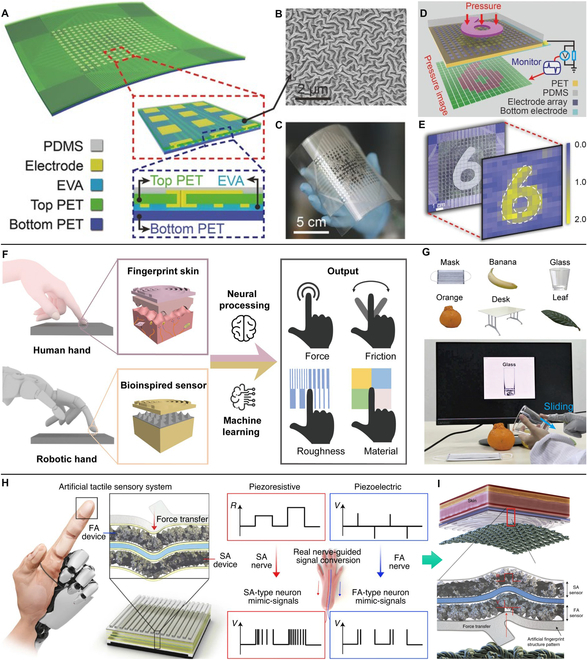
Tactile sensing devices of intelligent identification for VR applications. (A to E) Schematic illustration of a 16 × 16 pressure-sensitive triboelectric sensor matrix (A), SEM image of the etched PDMS surface microstructure (B), photograph of the sensor matrix (C), demonstration of the mapping output voltage of the sensor matrix under patterned pressure (D), and simulation results for the potential distribution (E). Reproduced with permission from [114]. Copyright 2016, John Wiley and Sons. (F and G) Schematic illustration of the biomimetic tactile system via a stick-slip sensing strategy (F) and the real-time object recognition system (G). Reproduced with permission from [116]. Copyright 2022, Springer Nature. (H and I) Schematic illustration of the artificial neural tactile sensing system (H) and the sensor responding to textured object (I). Reproduced with permission from [119]. Copyright 2021, Springer Nature.

As shown in Fig. 7A, Wang et al. [114] designed a pressure-sensitive triboelectric sensor matrix and realized real-time tactile mapping. They treated PDMS with dry etching, which generated a micro/nanostructure surface (Fig. 7B) that could increase the sensing performance of the sensor. Figure 7C shows the flexible matrix of 16 × 16 pixel with a resolution of 5 dpi. Accordingly, patterned pressure (e.g., mold in a shape of “6” in Fig. 7D) imposed on the matrix could be readily imaged though the simultaneous use of multiple pixels (Fig. 7E), which would offer opportunities for application in VR-based HMIs. Based on biomimetic mechanoreceptor and stress field sensing, Shang et al. [115] reported an electronic skin for modular multi-parameter perception. It could decode complex tactile information into field information. By reconstructing and analyzing the stress field, distribution of 3D forces could be resolved with 1.8° polar angle resolution and 3.5° azimuthal angle resolution, and the hardness of object could also be detected.

With tactile sensors of multi-mode, the surface texture can also be recognized. For example, Li et al. [116] proposed a multifunctional biomimetic tactile system via a stick-slip sensing strategy, which was a universal approach for monitoring slippage and estimate the surface characters of objects by sliding. As is depicted in Fig. 7F, the sensor consisted of an epidermis-inspired double-helix top layer and a spinosum-inspired pyramidal bottom layer, which were designed for transmitting vibrations obtained from stick-slip phenomena and sensibilization, respectively. Assisted by deep learning, this system realized a high recognition rate of 100.0% for both static and sliding states, and obtained the ability to distinguish six types of materials (93.3%) and six different roughnesses (92.8%) (Fig. 7G).

Shan et al. [117] designed a photoelectric tactile sensing system for high-accuracy texture recognition. This system realized the simulation of human tactile information, and the recognition of 16 kinds of fabrics was distinguished with an accuracy of 94.1%. Chun et al. [118] reported a tactile sensor with a single sensor architecture made of single-layer graphene. Because of the local deformation of a specific area of the single-layer graphene, resistance variations could be reflected in the resistance of the entire sensor and the sensor was able to detect a vertical pressure as low as 24 Pa with a fast response of ~2 ms for deformation and ~3 ms for restoration. Thus, by introducing microstructures inspired by human fingerprints, surface texture was successfully defined through fast Fourier transform analysis, which provided a simple method that, using a single sensor, realized surface texture recognition at the level of human sensation. Chun et al. [119] reported an artificial neural tactile sensing system using particle-based polymer composite sensors (piezoresistive and piezoelectric) and a signal-converting system (Fig. 7H). The sensor could specially respond to pressure and vibration, and its output signals were similar to those of slow-adapt and fast-adapt mechanoreceptors. The system integrated with sensing signals and deep learning was able to classify fine and complex textures (Fig. 7I), and could also be used to predict unknown textures on the basis of the trained model.

**Fig. 8.**
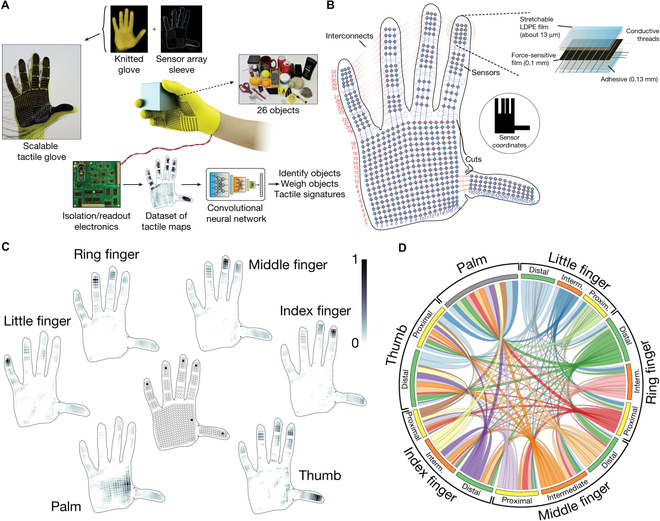
Tactile sensing devices of object identification for VR applications. (A to D) Schematic illustration of the scalable tactile glove for learning the signatures of human grasp (A), the individual locations of the sensor unit (B), the set of decomposed object-related pressure frames (C), and the circular plot of the relative correspondences between different parts of the hand (D). Reproduced with permission from [122]. Copyright 2019, Springer Nature.

Distinguishing object is also another intelligent application of tactile sensors [120]. Li et al. [121] integrated quadruple tactile sensors with a robot hand and realized precise object recognition via grasping. The quadruple sensor was fabricated with skin-inspired multilayer microstructure and worked as thermoreceptor and pressure sensor to perceive thermal conductivity of a material and the contact pressure, respectively. Combining data acquisition and processing, the intelligent robot hand was able to precisely recognize objects of various shapes, sizes, and materials, which was beneficial for VR system to realize complex recognition. In Fig 8A and B, Sundaram et al. [122] created a scalable tactile glove to help learn the signatures of human grasp. The glove was fabricated with piezoresistive pressure sensors to analyze the grasp patterns of human hand based on pressure distribution (Fig. 8C). As shown in Fig. 8D, they recorded a large-scale tactile map (a dataset of 135,000 frames) when grasping various objects with a single hand. Deep learning process revealed that the spatial correlations and correspondence between finger regions that emerged from the dataset represented the tactile signatures of the human grasping strategy. Thus, these tactile data were very useful for duplicating operations of virtual hand in a VR system.

### Machine learning in intelligent haptic sensing

In haptic sensing area, data acquisition and processing are gradually becoming the most important part since multimode and real-time sensing bring a bulk of data. As a main branch of artificial intelligence, machine learning possesses the ability to analyze, extract, and decode these complex data, providing opportunities to efficiently handle the data process. The four main tasks of machine learning are classification, regression, clustering, and dimension reduction based on related algorithms. Machine learning applied in above haptic sensing devices is summarized in Table 2.

**Table 2.** Summaries of machine learning in haptic sensing device

Category	Reference	Algorithms	Task	Application	Accuracy
Piezoresistive	[26]	CNN	Classification and regression	Body motion recognition	63.76%
	[68]	MLP	Classification	Hand trajectory	98.8%
	[93]	SVM	Classification	Hand gesture identification	92.67%
	[94]	RBFNN	Classification	Hand gesture identification	93.3%
	[100]	CNN	Classification, clustering, and dimension reduction	Gesture recognition	96.7%
	[104]	RSL	Classification and regression	Human action recognition	96.2%
	[107]	CNN	Classification, cluster, and dimension reduction	Object identification	96.9%
	[116]	XGB	Classification	Material recognition	93.3%
	[122]	CNN	Classification, regression, clustering, and dimension reduction	Object identification	89.4%
Piezoelectric	[119]	-	Classification	Texture recognition	99.1%
Triboelectric	[88]	1D-CNN	Classification	Body motion recognition	96.67%
	[101]	SVM	Classification and feature extraction	Hand gesture identification	98.63%
	[83]	1D-CNN	Classification	Body movement trajectory	85.67%
	[86]	ANN	Classification	Gait signal identification	-
	[89]	CNN	Classification	Human action recognition	91.47%
	[106]	1D CNN	Classification	Body motion recognition	97.5%
	[112]	LDA	Classification	Object identification	96.8%
	[113]	VGG	Classification	Material recognition	96.62%
Hybrid (piezoelectric and triboelectric)	[96]	CNN	Classification	Hand gesture identification	94.16%
	[102]	LDA	Classification	Human action recognition	92.6%
Magnetic	[82]	-	Classification, clustering, and dimension reduction	Human action recognition	-
Electromyography	[103]	AM	Dimension reduction	Hand gesture identification	97.12%
Photoelectric	[117]	ANN	Classification	Texture recognition	94.1%
Thermal conductive	[121]	MLP	Classification	Object recognition	94%

## Haptic Feedback Techniques

For a closed-loop tactile interaction in VR system, haptic feedback is a reverse process compared with sensing devices. In the development of VR technology, visual, auditory, and olfactory have been maturely applied, while virtual tactile is still a weak link, because it needs feedback interfaces conformal with skin and the flexible, high resolution, and scalable characters are necessary. To date, efforts in haptic feedback exist, including techniques such as mechanical vibration [123], ET [124,125], DEA [126], and so on [127–129], which have enriched VR applied in immersive games, virtual communication, and teleoperated robotics.

### Mechanical vibration feedback

**Fig. 9.**
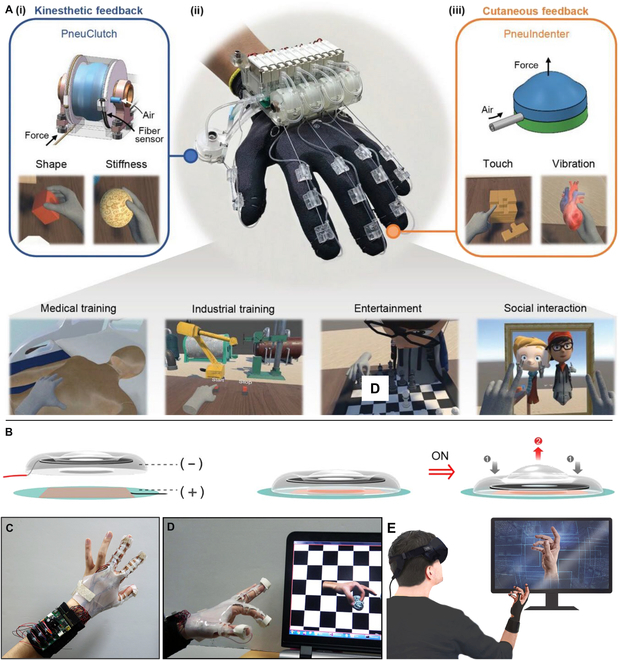
Haptic feedback devices of pneumatic actuator for VR applications. (A) Schematic illustration of the pneumatic glove (ii) consisting of PneuClutch (i) and PneuIndenter (iii) and its various applications. Reproduced with permission from [133]. Copyright 2023, John Wiley and Sons. (B to E) Working principle (B), photograph (C), operation image (D), and the promising VR system of the pneumatic actuator with fingertip feedback. Reproduced with permission from [134]. Copyright 2019, Springer Nature.

Mechanical vibrations, aiming to evoke the vibration-sensitive skin receptors, are able to generate virtual haptic sensations on skin. Vibration stimuli can be realized by pneumatic and magnetic actuators. Pneumatic actuators are always facilitated with soft membrane that can expand or contract relaying on air inflation or deflation, leading to the vibration stimulus [130,131] or fingertip force feedback [132]. For example, Qi et al. [133] reported a pneumatic glove shown in Fig. 9A. Two main modules were low-pressure (<60 kPa) actuated PneuClutch and PneuIndenter, which allowed this glove with untethered lightweight characters (283 g) and enabled users to sense kinesthetic and cutaneous feedback that realized touching, pressing, grasping, squeezing, and pulling virtual objects with immersive haptic sensation. The authors also developed a VR environment for using in object grasping and complex archery game with the glove, showing its potentiality in medical training, industrial training, entertainment, and social interaction. As shown in Fig. 9B, Song et al. [134] developed a pneumatic actuator to generate fingertip feedback. The integrated glove (Fig. 9C) used electrostatic force to generate inner air pressure instead of the bulky compressor, which makes it a light weight (0.57 g) and miniaturization system. Thus, the glove was capable of providing realistic feedback when holding a virtual object (Fig. 9D), contributing effective VR experiences conjugated with other virtual devices (Fig. 9E). Sonar et al. [135] provided a soft pneumatic actuator skin with soft strain sensors where high-frequency sensing and actuation were realized. The liquid metal sensor was embedded in the upper layer of the vesicular actuator to form a sensor and actuator laminate skin that could provide controllable actuator shape in real time up to 100 Hz at output forces up to 1 N. This bifunctional actuator skin offered a promising direction for intuitive and comprehensive haptic interactions. These pneumatic actuators induced controlled air flow to inflate or deflate the deformable bladder realizing skin stimulation. Although they require externalized hardware for pumping, the actuator merits its flexible structure to be conformal with human skin, and the soft membrane coordinated with appropriate air pumping can provide flexible and controllable virtual tactile sensation.

**Fig. 10.**
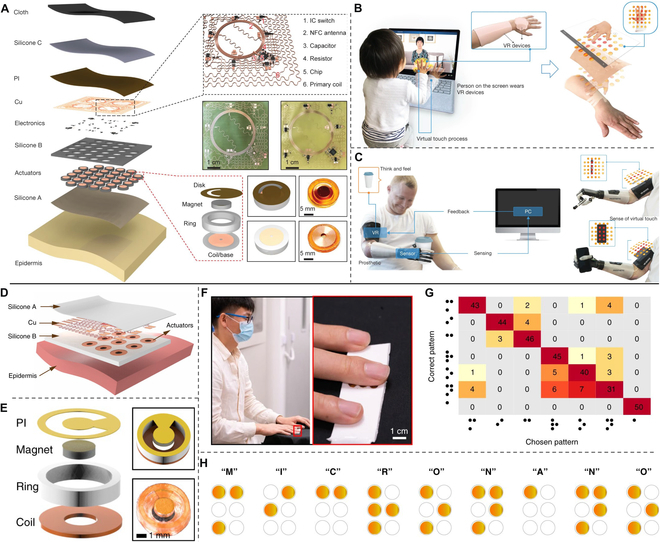
Haptic feedback devices of magnetic actuator for VR applications. (A to C) Exploded-view schematic illustration of the wireless haptic interface (A) and its applications demonstrated in social media interactions (B) and prosthetic feedback (C). Reproduced with permission from [136]. Copyright 2019, Springer Nature. (D to H) Exploded view of the fingertip interface (D), the exploded actuator (E), photograph of the interface integrated on finger (F), and the correction (G) of blind test to recognize Braille patterns (H). Reproduced with permission from [137]. Copyright 2021, Springer Nature.

Magnetic actuator is another mechanical tactile approach that is commonly used because of fast response and controlled flexibility. It relies on the vibration of miniature motors to generate sensations like object moving in a direction perpendicular to skin, which is able to simulate the most realistic haptic sensations. As shown in Fig. 10A, Yu et al. [136] reported a haptic interface with skin conformability, wireless, and battery-free characters, which could simulate skin by localized mechanical vibrations to create virtual haptic information of programmable patterns. The actuator is made of magnet actuators embedded in silicone-based substrate with controller of near-field communication (NFC) antenna. It could be directly laminated onto curved skin owing to the flexible encapsulation, and the actuator was capable of generating vibration force of about 135 mN with a fast-responding frequency (about 300 Hz) that was sufficient to simulate the skin. Figure 10 (B and C) demonstrates its applications in social media interactions and prosthetic feedback, and it was expected to offer opportunities in personalized rehabilitation, surgical training, and multimedia entertainment experiences. Li et al. [137] miniaturized this kind of actuator and developed a finger-integrated haptic interface (Fig. 10D and E). The actuators were designed in diameter of 5 mm and thickness of 1.45 mm to integrate a 2 × 3 array as dynamic Braille on fingertip (Fig. 10F). Figure 10G summarizes the correction of a subject group (five testers) in 10 groups of blind tests (totally 50 sets of data) when testing Braille recognition of patterns in Fig. 10H. For these seven letters, the average recognition accuracy was 85.4%, which was sufficient for Braille interaction. In magnetic actuator, the magnet can freely vibrate in an encapsulated interface so that the vibration intensities and high frequency are available controlled under current magnitudes, pulsation character, and oscillation frequencies.

Controllable frequency in a wide range is the advantage of mechanical vibration feedback, but also brings the nonconformal contact with the skin. There are developed adhesive interfaces to tackle this interface issue. For example, Kim et al. [138] reported a bio-inspired skin-attached haptic interface with interconnect structures that could resist sweating and vibration. This interface was designed with microchannels coated with a soft gel inspired by free frog and interconnected architectures inspired by snail, which provided with an attachment weighing about 300 g on rough sweaty skin and the reversible adhesion that could tolerate vibration-induced fatigue on both dry and sweaty skin surface. Integrated with a vibrational haptic actuator (10 mm shaftless vibration motor), the developed haptic interface could implement virtual physical interaction by recognizing human motion and vibrohaptic feedback. Hwang et al. [139] proposed adhesive interface for adaptive haptic interaction. Inspired by diving beetle-like small dense hairs possessing concave cavities, this interface obtained high adaptability on various nonflat surfaces with robust adhesion in dry (≈16 kPa) and wet (≈27 kPa) conditions. Integrating electronics with PVC gel actuator, the adhesive platform was able to transmit vibration to the skin surface. They encoded various vibration information to simulate the real surface texture of objects, and provided touching and feeling representative of a gecko lizard in a VR environment.

### ET feedback

Imparting appropriate electric current on skin is a straightforward approach to realize proprioceptive haptic feedback, since the skin receptors depend on bioelectricity to transmit tactile sensations [140–142]. The ET feedback devices are always fabricated with electrode pairs of anode and cathode to form the current flow under skin, and are advantageous for small size as well as high spatial resolution. Sato reported an ET display for the distribution of force vectors [143]. Since the selective stimulation of mechanoreceptors represented various distributions of force vectors, anodic and cathodic stimuli were induced to trigger FA receptors and SA receptors, respectively. The distributed force vectors were reproduced by selecting different stimulating point. Thus, this strategy indicated that the representation of the distribution of force vectors was feasible via ET stimulations. Komurasaki et al. [144] reported an electrovibration and electrical stimuli integrated haptic display based on micro-fabrication process. The frequency-dependent relationship of tactile perception toward electrovibration and electrical stimuli was revealed. Accordingly, virtual tactile sensations of vibrational friction, pressure, and vibration could be provided.

**Fig. 11.**
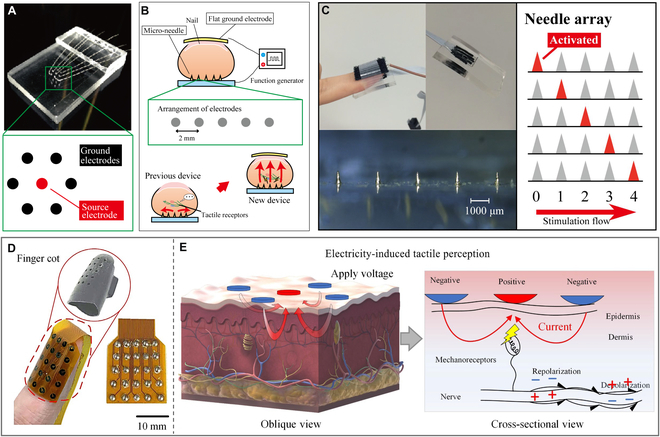
Haptic feedback devices of ET interface for VR applications. (A to C) ET interface made of micro-needle electrodes (A) and the arrangement of the electrode (B) and test image of the ET interface on fingertip (C). Reproduced with permission from [145]. Copyright 2016, PLOS. (D and E) Photograph of the ET electrode of the rendering system (D) and ET stimulation mechanisms with negative and positive current (E). Reproduced with permission from [146]. Copyright 2022, the American Association for the Advancement of Science.

For ET feedback, the appropriate current and voltage is the most concerning factor because of the drift skin impendence and variable individual properties. Tezuka et al. [145] improved the ET electrodes using a micro-needle electrode array (Fig. 11A). They arranged the needle electrodes on the fingertip and the ground electrode on the fingernail (Fig. 11B). The micro-needle electrode penetrated the part of stratum corneum but not reached the pain point. In this case, the high impendence of stratum corneum was overcame and the threshold voltage on fingertip was reduced under 10 V, which was smaller than that of plat electrode (over 70 V). The patterned ET stimulation was realized by the electrode array with a resolution of 2 mm (Fig. 11C). As shown in Fig. 11D and E, Lin et al. [146] proposed an ET rendering system realizing ET stimuli with both high spatial resolution (76 dots/cm^2^) and rapid refresh rates (4 kHz) based on a current-steering super-resolution stimulation technique. They used a high-frequency modulation approach to reduce voltage under 13 V, and achieved Braille display as well as digital virtual experiences.

**Fig. 12.**
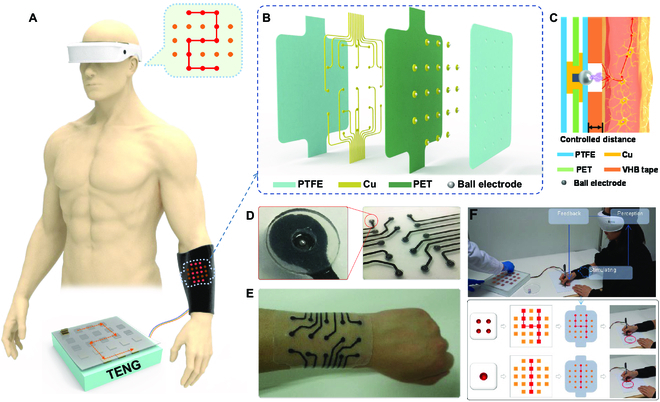
Haptic feedback devices of ET interface based on electrostatic discharge. (A to F) Schematic illustration of the ET system to transmit virtual spatial pattern (A), exploded view of the electrode array (B), ET sensation based on electrostatic discharge (C), photograph of the flexible ET interface (D) integrated on arm (E), and the application of virtual tactile interaction (F). Reproduced with permission from [147]. Copyright 2021, the American Association for the Advancement of Science.

To reduce current flow, as shown in Fig. 12A, Shi et al. [147] designed a self-powered ET system based on triboelectric nanogenerator and flexible ET interface. The ET interface was fabricated with ball-shape electrode (Fig. 12B), and the electrostatic discharge between electrode and skin could induce sensitive ET stimulation (Fig. 12C). They used the controlled distance to regulate the current induced into skin, resulting in an ultralow current of 25 μA, which was remarkably lower than the safety current threshold current (10 mA) on skin [148]. Via the effective ET stimulation with the skin-conformable interface (Fig. 12D and E), the virtual tactile interaction was realized in Fig. 12F, where the patterned hand input could be converted into electrostatic discharge on skin, and then sensed with the subject with right feedback. This ET system proposed a different ET approach than traditional ET devices and could be utilized in dynamic Braille, virtual tactile interaction, and augmented virtual protection. These strategies of micro-needle electrode and stimulation of electrostatic discharge solved the weakness of ET devices suffering from shifted impendence of human skin. The ET stimulation has advantages of simplicity and immediate ability with stretchable patterned electrode that facilitate integration with human skin to develop wearable virtual tactile devices.

### DEA feedback

**Fig. 13.**
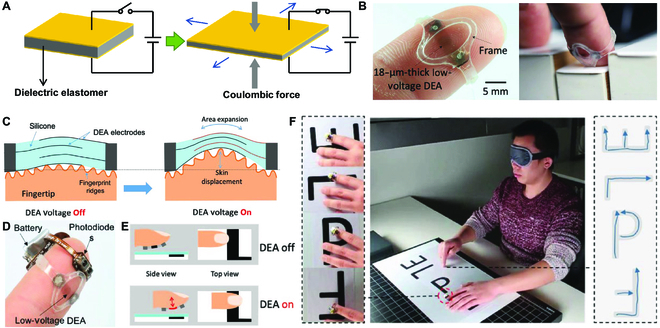
Haptic feedback devices of DEA for VR applications. (A) Principle of DEA. (B to F) Photograph of the feel-through DEA film in fingertip (B), working principle (C), photograph of the haptic reading system with photodiode as a swatch (D), reading principle (E), and tactile reading demonstration (F). Reproduced with permission from [151]. Copyright 2020, John Wiley and Sons.

DEA consisted of electroactive elastomer sandwiched between two compliant electrodes. When an appropriate voltage is applied, as shown in Fig. 13A, the generated attractive coulombic force will induce compressive stress on the elastomer, resulting in the deformation of the actuator, and the moderate deformed actuator is able to squeeze the local skin leading to tactile sensations. Koo et al. [149] developed a haptic display device that could offer stimulation on skin without any additional electromechanical transmission. The display was fabricated with electroactive polymer and flexible encapsulation, which could be directly integrated on fingertip. Mun et al. [150] presented a soft actuator based on multi-layered accumulation strategy. The layer-by-layer structure of electroactive polymer and electrodes provided the maximum vertical protrusion of 350 μm, and the output force was up to 255 mN, which was sufficient to stimulate skin. This large deformation also offered opportunities for realizing hierarchical ET stimulation, and five-level stimuli on forearm could be sensed by the subject. As shown in Fig. 13B, Ji et al. [151], using an 18-μm-thick DEA of multilayer structure, realized untethered feel-through haptic sensation. They integrated DEA, electronics, and battery into a haptic device of 1.3 g that is beneficial for wearing during everyday activities for extended periods. The DEA (with three electroactive layers) could provide stretches and compresses at frequencies from 1 to 500 Hz, and deform the skin in normal direction by over 6 μm (Fig. 13D). The demonstration in Fig. 13E and F was a tactile reading scenario, where the blindfolded subject could read the black letters using a photodiode to trigger DEA in the black area (Fig. 13F). Thus, this feel-through DEA device was able to be combined with visual and auditory device to realize more immersive VR experiences.

**Fig. 14.**
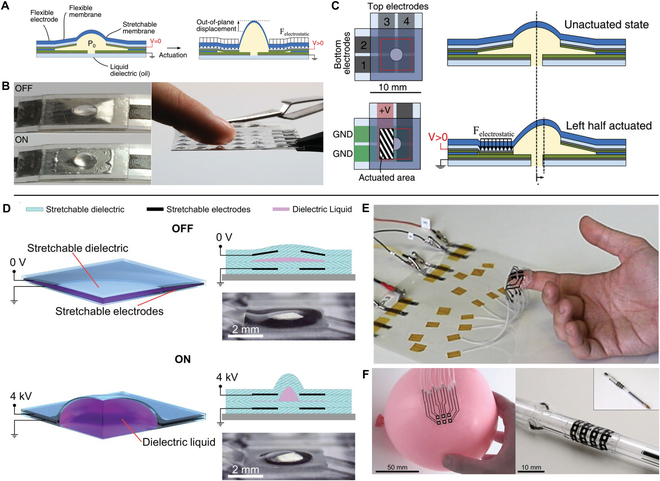
Haptic feedback devices of electrohydraulic actuator for VR applications. (A to C) The principle of the submillimeter-thick electrostatic actuator (A), photograph of off/on state (B), and illustration when powered by segmented electrode to generate in-plane forces (C). Reproduced with permission from [155]. Copyright 2020, John Wiley and Sons. (D to F) Schematic of the 3D-printed flexible haptic interface (A), photograph attached on finger (E), and curved surfaces (F). Reproduced with permission from [157]. Copyright 2023, John Wiley and Sons.

Electrohydraulic actuators are soft liquid-filled shells that deform due to electrostatic forces, which is indeed a variation of DEA [152–154]. Leroy et al. [155] reported a submillimeter-thick flexible hydraulically amplified electrostatic actuator that was able to deform out of plane and in plane, which could induce normal and shear forces to fingertip, hand, and arm. As shown in Fig. 14A, the actuator consisted of an oil-filled cavity with a metalized polyester boundary and a central elastomer region. When applying voltage to the annular electrodes, the dielectric oil could be squeezed into to the central stretchable region (Fig. 14B), generating a raised bump. Via segmented electrodes, the central bump could be pushed up, and also shifted north/south and east/west, forming rotation-like mechanostimulation (Fig. 14C). A 90-mg actuator in 6 mm × 6 mm could generate 300-mN force and displacements of 500 μm in a low-profile geometry (60% vertical strain). Owning to its thin structure and skin compliance, this actuator was promising for integrating a glove interface used in multimedia VR systems. Frediani et al. [156] used electroactive elastomer to control the liquid-filled bubble interface and realized tactile stimuli. The active dielectric elastomer would expend the bubble to release the liquid pressed on finger, while the maximum pressure occurred when voltage was off. The DEA was set to avoid any direct contact between skin, so that could provide electrical safety. Grasso et al. [157] developed a fully 3D-printed flexible haptic interface shown in Fig. 14D. The actuator was printed in six layers with dielectric oil filled in the cavity, and it depicted sufficient flexibility that could be directly attached to skin (Fig. 14E) as well as other curved surfaces (Fig. 14F). Even under stretch over 50%, the actuator could also give cutaneous stimuli at a wide frequency range (from DC to 1 kHz). By integrating a 2 × 2 array on fingers of human subject, the accuracy to recognize quadrant could reach 86%. Park et al. [158] proposed a soft haptic actuator based on knitted PVC gel fabric. Meriting from the intertwined structure, the electric field-induced deformation and electrostatic force are combined to maximize the vibration force and response time. Thus, the effects generated vibrations that were sufficiently strong for human perception. For these kinds of DEA actuators, they could directly convert electric signal into mechanical vibration without moving unit, avoiding bulk and mechanical complexity than mechanical vibration feedback. Although high input voltage is indispensable, its integration with dielectric liquid also provided shifted force stimulation, which impart DEA actuators multi-functions in haptic VR interactions.

As summarized in Table 3, examples of haptic feedbacks mentioned above are compared according to the mechanism, material section, and input and output properties. Subcategory of the main approaches is also listed in terms of the power mechanism, such as the pneumatic and magnetic actuators arranged clearly. The fabrication dimensions are noted with the shape of the device: dimeter (*D*), length (*L*), thickness (*T*), and weight (*W*). For mechanical vibration and DEA, the output parameters mainly concentrate on the none-load displacement (*D*), blocking force (*F*), and frequency (*f*), while ET devices merit for the output current (*I*) and high resolutions, which originate from their power source and point electrodes. Performance of pneumatic actuators depends heavily on the input air pressure, while that of magnetic actuator and DEA are decided by the input current signal covering voltage and frequency. Besides, typical applications of listed examples are also described. In summary, mechanical vibration are advantageous for the high safety and available frequency range, and ET devices with the point electrode and light weight are favorable for wearable designing and high resolutions. Although DEA needs high voltage to trigger virtual tactility, its flexibility and skin-compliant property are worthy of more attentions.

**Table 3.** Examples of haptic feedback devices summarized with their mechanism, material properties, design features, and input and output characters. Reproduced with permission from [183,185]. Copyright, John Wiley and Sons and Springer Nature.

Approach	Key mechanism	Reference	Materials and characters	Dimensions (mm)/weight	Array	Position	Output parameters (voltage/current)(displacement/force/frequency)	Resolution	Input (electric/mechanical signal)	Demonstration/application
Mechanical vibration	Pneumatic actuator	[130]	Air chamber created with silicone layers	*D* = 15*T* = 1.4–1.5	3 channels	Fingertip	*F* = 0.3 N*f =* 5–100 Hz	10 mm	Pressure, 70 kPa	-
	Pneumatic actuator	[132]	PDMS membrane	*L* = 22*T* = 8	-	Fingertip	*D* = 6 mm*F* = 1 N*f* = 0.1–10 Hz	-	Pressure, 20 kPa	Virtual control
	Pneumatic actuator	[133]	PDMS/Ecoflex	*D* = 9*T* = 1.5	-	Fingertip	*F* = 3.23 N*f* = 10–160 Hz	-	Pressure, 100 kPa	Virtual control
	Pneumatic actuator powered by electrostatic force	[134]	Ecoflex	*D* = 15*T* = 5*W* = 0.57 g	-	Fingertip	*D* = 0.15 mm*f* = 0.2, 0.3, 1.0 Hz	-	Square wave, 0–6 kV	Virtual control
	Pneumatic actuator	[135]	Silicone elastomer	*D* = 10	-	Fingertip	*D* = 5 mm*f* = 0–100 Hz	-	Pressure, 30 kPa	-
	Flexible voice-coil magnetic actuator	[136]	Cu coil, NdFeB magnet with PDMS encapsulation	*D* = 18*T* = 4.0*W* = 1.4 g	32 channels	Arm, bac, opisthenar	*D* = ≈191–300 μm*F* = 135 mN*f* = 100–300 Hz	18 mm	Pulse, 5 V	Social media communication, feedback prosthetic, immersive games
	Magnetic actuator	[137]	Cu coil, magnet with PDMS encapsulation	*D* = 5*T* = ≈1.8	3 × 3	Fingertip, wrist	*D* = ≈1.55 mm*f* = 200 Hz	~5 mm	Sine wave, 0.01–0.5 V, 1.12–56.18 mA, 70–740 Hz, 1.4 mW	Dynamic Braille
Electrotactile	Electrical stimuli	[141]	Steel electrodes	*D* = 1	4 × 4	Fingertip	Pulse, 0.2 ms,I = 1.0–3.0 mA*f* = 30 Hz	2–4 mm	Pulse, 0.2 ms, 100–300 V, 1.0–3.0 mA, 30 Hz	-
	Electrical stimuli	[143]	Pin electrode (Cu)	*D* = 1	31 channels	Fingertip	Pulse, 20 μsI = 0.0–5.0 mA*f* = 210 Hz	2 mm	Pulse, 20 μs0.0–5.0 mAf = 210 Hz	Force vector identification
	Electrovibration and electrical stimuli	[144]	Cr electrode	18 × 0.9	-	Fingertip	I = 0–5 mA,*f* = 5–320 Hz	2 mm	0–600 V, 0–5 mA, 5–320 Hz	-
	Electrical stimuli	[145]	Microneedle electrode (Ti)	*D* = 0.3	1 × 5	Fingertip	*f* = 10–100 Hz	2 mm	10–100 V, 10–100 Hz	-
	Electrical stimuli	[146]	Ag/Cu electrode	*D* = 1	5 × 510 × 10	Fingertip, palm	*f* = 10 kHz	4 mm	Square wave,15–20 V, 10 kHz	Font Braille, electrotactile rendering
	Electrostatic discharge stimuli	[147]	Tin ball-shaped electrode	*D* = 0.5	21 channels	Fingertip,forearm	I = 25 μA	3 mm	Kinetic energy, body movement	Social media communication, dynamic Braille
Dielectric elastomer actuator (DEA)	DEA	[149]	8-layer silicone layer separated with flexible carbon electrode	*D* = 2*T* = 2	4 × 5	Fingertip	*D* = ≈471 μm*F* = 14 mN	3 mm	Square wave, 3,500 V, 0–100 Hz	-
	DEA	[150]	6-layer silicone layer separated with flexible Ag NW electrode	*D* = 16*T* = 0.7	2 × 3	Fingertip, forearm	*D* = ≈300–650 μm*F* = 50–250 mN	-	Sinusoidal wave, 3–4 kV, 0–500 Hz	-
	DEA	[151]	PDMS membrane with PMMA holder, carbon nanotube	*T* = 18 μm*W* = 1.3 g	-	Fingertip	*f* = 1–500 Hz	-	480 V	Haptic reader
	Electrohydraulic actuator	[153]	Dielectric oil, silicone membrane, dielectric shell and Ag electrode	*D* = 2	7 channels	Fingertip, palm	*D* = 500 μm*P* = 1 MPa	3 mm	0–4 kV	Feedback recognition of surface texture and object shape
	Electrohydraulic actuator	[154]	Dielectric oil, stretchable membrane, ITO electrode on PET holder	*T* = 500 μm*L* = 22	16 channels	Fingertip	*D* = 1.5 mm*F* = 4 N*f* = 2 Hz	-	1–1.5 kV, 0–30 Hz, 6 mW	Feedback touchscreen
	Electrohydraulic actuator	[155]	Dielectric oil, PDMS membrane and aluminum electrode	*L* = 10	5 × 5	Fingertip, arm	*D* = 500 μm*F* = 300 mN	10 mm	Square bipolar signal, 0–1,400 v, 200 Hz	Normal and shear force feedback
	Electrohydraulic actuator	[156]	VHB dielectric film, silicone/carbon black composite electrode and insulating silicone grease	*D* = 12.5*T* = 5.5	-	Fingertip	*F* = 1 N	-	Square wave, 0.5–4 kV, 0.1–3 Hz	Various feedback identification
	Electrohydraulic actuator	[157]	Dielectric oil, carbon black electrode, PDMS encapsulation and PET substrate	*T* = 350 μm*W* = 1 g	2 × 3	Fingertip	*D* = 200 μm*F* = 40 mN	-	Sine waves, 4 kV, 1–1,000 Hz	Static haptic stimulation

### Other feedbacks

**Fig. 15.**
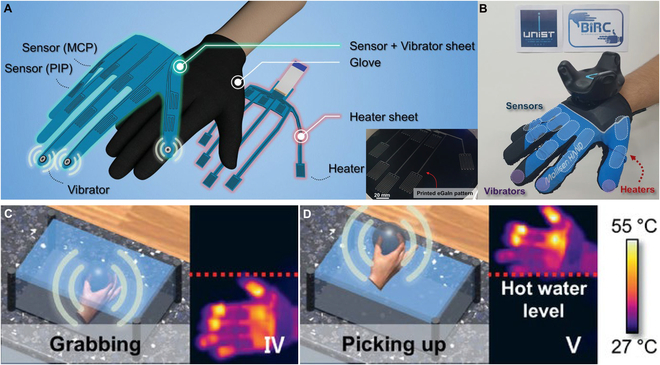
Thermal feedback devices for VR applications. (A and B) An exploded view of the thermal haptic interface (A) and integrated haptic glove (B). (C and D) Virtual scenarios when grabbing (C) and picking up (D) a metal ball and real-time temperature images by infrared camera. Reproduced with permission from [172]. Copyright 2020, John Wiley and Sons.

In addition to mentioned approaches, other novel feedbacks have been investigated for expanding applications of haptic feedback in VR systems, such as thermal feedback [159,160], ultrasound feedback [161,162], and piezoelectric actuator [163]. Temperature sensing is also considered as the main function of human tactile receptor, and temperature sensation originates from heat flux occurring when contacting with object of different thermal conductivities. Various thermoreceptors under skin are able to detect independent temperature zones with a wide temperature range of 4 to 52 °C and resolution of 0.02 °C [164]. There are Joule heating [165], electrocaloric effect [166], magnetocaloric effect [167], and thermoelectric effect [168] that are induced to realize virtual thermal sensation, and functional materials, such as fluidic heat transfer [169], mechanocaloric materials [170], and phase-charge materials [171], can also generate varied heat exchange with skin. Oh et al. [172] developed a thermal feedback device based on patterned liquid metal (Fig. 15A) and integrated glove (Fig. 15B). The printed liquid metal electrode was encapsulated in silicone layers and has improved heating performance from 25 to 85 °C even strained to 50%. They finally proposed a multimodal sensing and feedback system that enabled immersive VR experiences (Fig. 15C and D). Lee et al. [168] reported a skin-like wearable thermo-haptic device that could actively cool down and heat up deformable skin surfaces even under maximum stretching over 230%. The unique highlight of this haptic interface was the capability to rapidly return to the original temperature after reaching the target temperature within a few seconds by changing the direction of current, which was beneficial for the fast thermal sensation without subsequent unintended parasitic thermal residue that enabled to realize various virtual situations with higher accuracy. Li et al. [173] integrated a thermal and vibrational feedback glove based on miniature vibrators and semiconductor refrigerators. It could allow temperature feedback change from 10 to 47 °C, and realized the VR experience to dilute sulfuric acid in a virtual environment. Thermal haptic feedbacks as an approach to realize virtual haptic over mechanical force and vibration will enhance the degree of artificial immersion and render VR/AR in a more authentic manner. Ultrasound feedbacks, relying on mid-air interface, are also promising approaches because they allowed users to avoid being tethered to any holdable or wearable devices. For example, Howard et al. [174] reported a pan-tilt ultrasound interface for larger interaction workspace in VR. It could provide delivering sensations from multiple different directions because of two degrees of freedom, enlarged usable workspace in a 14-fold increase, focal point repositioning speeds over 0.85 m/s, and positional accuracy below 18 mm. Liu et al. [161] used a piezoelectric mircromachined ultrasonic transducer (pMUT) to achieve ultrasonic haptic feedback. This pMUT is able to provide resonance frequency of 32.9 kHz and unit acoustic pressure of 0.227 Pa. The integrated array of 251 × 251 pMUTs enabled sufficient acoustic pressure to create haptic sensations, and this proposed demonstration has potential to be applied in ultrasound haptic feedbacks.

## Applications

The development of haptic sensing and feedback devices has promoted the innovation of VR techniques and also provided more immersive experiences than systems only with visual and auditory interactions. Haptic sensor acts as an input approach, and feedback offers available output modality, which have enhanced the multi-functionality of VR systems [175–177].

**Fig. 16.**
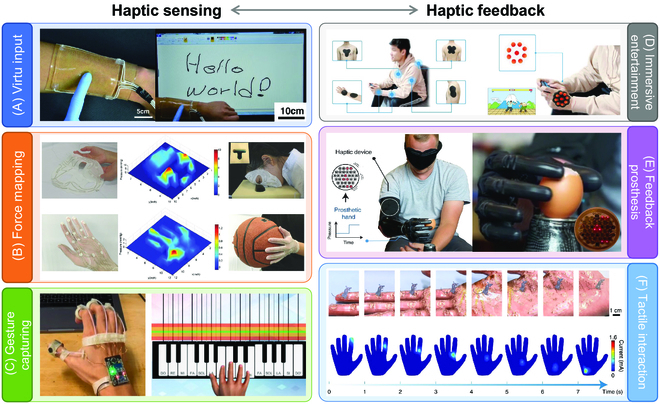
VR applications of haptic sensors and feedback devices. (A to E) Application examples of haptic sensors in virtual input (A), force mapping (B), and gesture capturing (C), as well as haptic feedbacks in immersive entertainment (D), feedback prosthesis (E), and tactile interaction (F). Reproduced with permission from [7,136,178,179,181,183]. Copyright, the American Association for the Advancement of Science, John Wiley and Sons, and Springer Nature.

Haptic sensors are mostly used for collecting touch information including contact or not, pressure quantity, and force distribution, which are important data for modelling in virtual world. For example, Kim et al. [178] reported a soft transparent touch panel based on ionic hydrogel, and applied it as an epidermal input panel that realized virtual input (Fig. 16A). As shown in Fig. 16B, Li et al. [179] introduced a flexible supercapacitive nanotexture that achieved pressure monitoring such as facial and gripping pressure mapping, which could facilitate the signal transforming modality for next-generation VR systems. Basically, to capture hand gesture is the first step to reconstruct hand-based task and give instructions in VR environment. For example, Sun et al. [7] used their ring sensor (intergrade on finger in Fig. 16C) to collect the data knuckle movements and processed the data to reproduce the hand instruction for playing a virtual piano. Also, capturing body motion is the advanced application of hand gesture recognition. Gong et al. [180] designed a piezoresistive artificial bionic skin that could adhere compliantly on human body to record the body motion, and demonstrated its application to capture motion gesture and wirelessly control a robot in real time.

Haptic feedback as the reverse process of sensing enables users to understand touch information such as materials, texture, and roughness, and proved immersive HMI experiences. Simultaneously, corresponding instructions made by users are able to enhance the control and collaborative ability in HMI, contributing to closed-loop HMI. Therefore, haptic feedback devices have been applied in immersive entertainment, feedback prosthesis, and tactile interactions. The wireless haptic interface, reported by Yu et al. [136], could be attached on opisthenar, arm, and back (Fig. 16D) and generated mechanical vibrations to simulate the impacts when playing combat game. Jung et al. [181] reported skin-integrated haptic actuators to facilitate the robotic prosthetics. As shown in Fig. 16E, the amputee could indirectly sense tactile experience of contact according to the vibration intensities and patterns and transmit instructions to the prosthetics. Thus, the eggshell could be grasped without broken shell. The application of prosthetic is similar as controlling virtual robotic arm in a VR environment. Fan et al. [182] designed a Digital Twin-driven mixed reality framework for immersive teleoperation with haptic rendering based on soft pneumatic haptic feedback, and realized immersive teleoperating in both real and realistic environment. The ET interface shown in Fig. 16F could transmit safe current to skin to generate ET sensations and could translate the trajectory of virtual mouse into tactile patterns to achieve tactile communications from virtual environment to real world [183]. Thus, haptic feedback process in VR system is more of immersion than that only with vision and auditory environments. For example, the vibration of cell phone would increase shock sensation when playing phone games. Accordingly, such skin-integrated vibration feedback can provide mimic impact during fighting game, and to be vagarious, an overdosage ET stimulation is able to reproduce pain sensation when playing shooting games. This haptic feedback can be extended to be applied in virtual military training to increase the immersive level.

In addition, the requirement of artificial intelligence of things (AIoT), wise information technology of medicine (WITMED), and smart home and cities will create promising paths forward for haptic sensing and feedback. The virtual control of teleoperated robot is an example that haptic sensing and feedback are necessary. The human hand instructions need to be translated into control code by sensing devices that are also in charge of monitoring the operation status between robotic hand and object. The feedback section is also important, because it makes the operator in real time aware of the operation status and gives following instructions. The integrated sensing and feedback are especially a key point for special task with fragile object. Surgery simulation and training also need virtual haptic interactions to allow doctors to perform in virtual environment. Haptic feedback can provide sensations similar with the real process to accomplish the training purpose. This is also applied for the interactive education in which students are capable of understand the learning content and experimental subject, which can enhance the teaching in an immersive way. In entertainment area, virtual vision and audition have been realized with VR glasses and audio devices, but virtual haptic is missed. Inducing body sensing and feedback parts, for example, the body gesture capturing and force feedback, will greatly improve the levels of immersion. To expect that if a haptic feedback servicer covering data of hand shaking and hugging is established, the multi-media social communication maybe come true. In this case, haptic greeting is not impossible to realize without the special and temporal limits. The greeting can be sent to ourselves in tens of years future, which will be an obsessed experience.

## Conclusions and Prospects

The past decade has seen the rapid development of haptic sensing and feedback devices along with efforts in emerging materials, miniaturized electronics, and data processing, and their applications range from HMI to medical monitoring, interactive entertainment, immersive learning, and remote surgery. However, challenges in this area still remain. For haptic sensing devices, first, the sensitivity and accuracy need to be improved. Most of the time, the wide liner responsibility and high sensitivity cannot be realized simultaneously because they are heavily dependent on the modulus character of active materials. Next, stability and durability of the wearable devices should be guaranteed during exercising, sweating, or other operating circumstances that are extremely cold or hot. Finally, technical conflicts between data processing and real-time response exist, because the time to finish an operation (e.g., hand gesture) is necessary, and so is it to collect the data for recording the operation. Thus, the data processing is always delayed, resulting in an asynchronous HMI process. Besides, the most developed haptic sensors are still under laboratorial level and disjointed with realistic applications. The integration between wearable sensor and VR system is still in a low degree due to the complex data acquisition, transformation, and hardware analysis.

For the mentioned kinds of haptic feedback approaches, they all suffer from technical bottlenecks. Mechanical vibrant feedback heavily relies on magnet of millimeter level, failing to integrate thin-film devices, and the high-resolution skin stimulations are always sacrificed. ET method, though proud of high resolution, is stuck in the appropriate current and voltage to adapt to the variable skin impedance across the body and between individuals. In terms of DEA, depending on the hundreds or thousands of voltages to power millimeters of deformations, the safety and sensitive skin stimulation cannot be guaranteed. In addition, the functions of these feedbacks are limited only in pressure feedback, and virtual haptics, such as slip sensation, thermal feedback, and cold sensation, are difficult to be realized. The high-resolution haptic stimulation needs to further increase the density of stimulators, while sophisticated wiring and signal transmitting are necessary. Most importantly, for the cooperation of haptic sensing and feedback, devices can hardly realize closed-loop virtual haptic interactions, which are supposed to be a future mainstream in developing VR systems. The commercializing development of haptic sensing and feedback devices is also a considerable challenge, because most devices in laboratory do not keep price in consideration, the cumbersome wired power source, data acquire meter, and processing hardware are necessary, the integrations of these devices with others of different mode (e.g., the visual part) are difficult, and the wearability in daily life is also under development.

However, the developments in haptic interaction are still expected and promising. Following are several specific trends and developments of haptic interaction in VR system.

a. From materials aspect, burgeoning graphene, 2D materials, liquid metal, and ionic gel will boost the transparent, flexible, self-healing, self-adopt, biocompatible characteristics of sensing and feedback interfaces.

b. Advanced workmanship, such as 3D printing, direct laser writing, electron beam lithography, and microelectronic packaging, provides opportunities to fabricate flexible electrode of various patterns with high conductivity, micro-structured active layers with increased sensitivity, miniaturized multi-mode array with multi-functions, and miniaturized wearable devices that are portable, self-powered, and skin-integrated.

c. Emerging computer technologies, for example, artificial intelligence and deep learning, are accelerating the data acquisition and processing segment. For example, this would decrease the difficulty to extract characteristic signal from interminable data, which will facilitate the gesture identification and virtual model establishment.

d. Multi-mode haptic feedback, not only in force feedback, but also containing slip sensation, thermal feedback, and cold sensation, will increase the diversified multi-media interaction between human and virtual environment.

e. Closed-loop haptic interaction process is the ultimate goal. Real-time haptic sensing and simultaneously feedback will be finally applied in virtual control, smart protheses, virtual surgery, and so on.

Challenges and opportunities of haptic sensing and feedback will coexist in the near future. Although haptic perception is a subjective and complex process, and haptic interaction is a multidisciplinary and interdisciplinary area, co-efforts have been dedicated to materials, electronics, mechanics, and computer science. We believe that the bottleneck in this area will be overcome, and haptic interaction in VR systems will benefit applications including but not limited to medical, education, rehabilitation, internet of things, robotics, entertainment, and so on.
